# The Association between Gut Microbiota and Depression in the Japanese Population

**DOI:** 10.3390/microorganisms11092286

**Published:** 2023-09-11

**Authors:** Yichi Yang, Mone Mori, Kyi Mar Wai, Tao Jiang, Yoshikuni Sugimura, Wataru Munakata, Tatsuya Mikami, Koichi Murashita, Shigeyuki Nakaji, Kazushige Ihara

**Affiliations:** 1 Department of Social Medicine, Hirosaki University Graduate School of Medicine, Hirosaki 036-8562, Japan; yangy@hirosaki-u.ac.jp (Y.Y.); kyimar@hirosaki-u.ac.jp (K.M.W.); h23gm129@hirosaki-u.ac.jp (T.J.); y.sugimura@hirosaki-u.ac.jp (Y.S.); nakaji@hirosaki-u.ac.jp (S.N.); 2 School of Medicine, Hirosaki University, Hirosaki 036-8562, Japan; 3 Department of Hematology, National Cancer Center Hospital, Tokyo 104-0045, Japan; wmunakat@ncc.go.jp; 4 Innovation Center for Health Promotion, Hirosaki University Graduate School of Medicine, Hirosaki 036-8562, Japan; tmika@hirosaki-u.ac.jp; 5 Center of Innovation Research Initiatives Organization, Hirosaki University, Hirosaki 036-8562, Japan; murasita@hirosaki-u.ac.jp

**Keywords:** gut bacteria, mental disorder, gut–brain axis, interleukin-6

## Abstract

Depression is a leading cause of disease worldwide. The association between gut microbiota and depression has barely been investigated in the Japanese population. We analyzed Iwaki health check-up data collected from 2017 to 2019 and constructed generalized linear mixed models. The independent variable was the relative abundance of each of the 37 gut microbiota genera that were reported to be associated with depression. The dependent variable was the presence of depression assessed by the Center for Epidemiologic Studies Depression Scale. Potential confounders, including grip strength, gender, height, weight, smoking, and drinking habits, were adjusted in the regression models. Nine genera’s regression coefficients (*Alistipes*, *Blautia*, *Coprococcus*, *Dorea*, *Faecalibacterium*, *Holdemania*, *Lactobacillus*, *Mitsuokella*, and *Oscillibacter*) showed statistical significance after multiple comparisons adjustment. Among these nine gut bacteria genera, *Alistipes*, *Blautia*, *Coprococcus*, *Dorea*, *Faecalibacterium*, and *Oscillibacter* were reported to be associated with butyrate production in the intestine. Our results indicate that gut microbiotas may influence the depression condition of the host via the butyrate-producing process.

## 1. Introduction

Major depressive disorder (MDD) is one of the most common psychiatric disorders characterized by a loss of pleasure and interest, as well as a persistent depressive mood [1]. Patients may also experience symptoms such as insomnia, fatigue, and other related manifestations. MDD is regarded as one of the most burdensome diseases globally [2]. Despite the implementation of appropriate treatments, the disease burden of MDD can only be reduced by approximately one-third [3]. Due to its significant impact on personal well-being and societal welfare, exploration of the etiopathogenic processes of depression is necessary.

Gut microbiota is a population of single-cell microorganisms that inhabit the intestines of humans and animals [4]. It has been discovered to be associated with various biological and pathological processes, including the development of diseases [5]. The interaction between gut microbiota and the central nervous system, known as the gut–brain axis, has emerged as a promising area of research. Exploring this axis holds the potential to shed light on the pathogenesis of neuropsychiatric disorders [6].

Previous studies have indicated that gut bacteria may be involved in the etiopathogenic process of depression through immune system activation in the intestine. It has been observed that patients with MDD have higher levels of the concentration of interleukin-6 (IL-6), a pro-inflammatory cytokine involved in immune homeostasis, in blood compared to the healthy population [7,8]. Systematic reviews have also found positive associations between depression and other blood inflammatory markers, such as C-reactive protein and interleukin-1 [9,10]. One hypothesis regarding the influence of inflammatory pathogenesis on MDD involving gut microbiota is that inflammation-induced gut microbiota translocation into intestinal epithelial accelerates the release of immune mediators, such as IL-6. Elevated levels of immune mediators may disrupt the regulation of the hypothalamic–pituitary–adrenal axis, leading to increased circulating levels of stress hormones and ultimately exacerbating depression symptoms [6]. Pro-inflammatory cytokines released into the bloodstream can transmit signals to the brain via receptors on endothelial cells, thereby impacting the central nervous system through the blood–brain barrier [11]. Another possible hypothesis is that short-chain fatty acids (SCFAs) mediate the relationship between gut microbiota and depression. SCFAs, including butyrate produced by gut microbiota, have been shown to benefit mental health [12]. The proliferation of SCFA-producing gut microbiota and increased levels of SCFAs may enhance the tightness of epithelial junctions, preventing the translocation of inflammatory gut bacteria species [6]. On the contrary, inflammation in the intestinal environment may contribute to alterations in gut microbiota [13]. Inflammation and SCFA production may be confounders that derive the association of gut microbiota with depression rather than being mediators. For instance, inflammation and SCFA production may influence both the abundance of gut microbiota and the severity of depression. Further research is needed to elucidate the roles of inflammation and SCFAs in the relationship between gut microbiota and depression.

Dietary habits are known to contribute to the composition of gut microbiota. Previous studies have reported that a high intake of soluble fiber can improve the abundance of gut microbiota that produce butyrate while reducing the levels of pro-inflammatory cytokines [14]. Dietary habits have also been linked to depression directly [15]. It remains unclear whether dietary habits act as confounders, modifying both depression status and gut microbiota abundance simultaneously in the relationship between gut microbiota and depression, or if dietary habits affect the condition of depression through their impact on gut microbiota.

A considerable number of studies have been conducted to investigate the association between gut microbiota genera and depression. For example, in an observational study, Chen et al. compared the gut microbiota of ten patients with MDD and ten healthy controls, revealing a significant difference in the abundance of *Prevotellaceae* in a fecal sample between the two groups [16]. Other observational studies have reported lower levels of *Faecalibacterium* and higher levels of *Oscillibacter* in patients with MDD compared to healthy subjects [17,18]. Animal testing has also been conducted using gut microbiota as an intervention. Researchers have confirmed that the transplantation of microbiota derived from MDD patients caused depression-like behaviors in mice [19,20,21]. However, the outcomes of previous studies lack consistency, and most of them have been conducted with small sample sizes. The majority of research has focused on the Chinese population, with limited studies targeting the Japanese population [13]. Additionally, prior studies rarely incorporated blood inflammatory markers, such as IL-6 or SCFAs, when analyzing the association between gut microbiota and depression.

By analyzing a large dataset from the Japanese population, the present study aims to achieve the following objectives: (1) To investigate the association between gut bacteria and depression, thereby providing further evidence regarding their relationship in the context of the Japanese population. (2) To examine whether the pro-inflammatory cytokine, IL-6, acts as a mediator between gut microbiota and depression. (3) To elucidate whether the intake of soluble fiber, which serves as a substrate for butyrate production, acts as a confounding factor in the association between gut microbiota and MDD.

## 2. Materials and Methods

### 2.1. The Iwaki Health Promotion Project

The current study has a longitudinal, observation design utilizing data from the Iwaki Health Promotion Project (IHPP). The IHPP is a large-scale health check-up initiative based in the local community, aimed at enhancing the quality of life [22]. The IHPP welcomes participation from all adults in the local community who are 20 years of age or older. Residents who had previously registered as participants and could independently attend the health check-up venue were included in the project. There are no extra exclusion criteria for sampling. Approximately 1000 citizens residing in Aomori prefecture, Japan, participate in the IHPP annually, contributing a wide array of data from 2005. This includes demographic characteristics, psychological questionnaire results, physical ability metrics, blood samples, dietary data, and more. Our team carried out a post hoc analysis on the data compiled by the IHPP from 2017 to 2019, resulting in 2192 observations from 1135 participants after rigorous data cleaning. 

### 2.2. Microbiota Composition Measurements

Regarding the gut microbiota composition measurement, we provided IHPP participants with fecal sample kits before the health check-up and asked participants to collect fecal samples at home. The fecal sample kits contained guanidinium thiocyanate solution (100 mM Tris-HCl (pH 8.0), 40 mM Tris-EDTA (pH 8.0), 4 M guanidinium thiocyanate, and 0.001% bromothymol blue) (TechnoSuruga Laboratory Co., Ltd., Shizuoka, Japan) to ensure the stability of the gut microbiota composition. Participants were instructed to store the fecal samples in their home refrigerator before they submitted the samples on the day the IHPP was held. After submission, the fecal samples were stored in a 4 °C environment at the Technosurga Laboratory for three months, awaiting DNA extraction. The bead-beating method was performed to extract DNA from the gut microbiota. The DNA purification process involved the use of an automated nucleic acid extraction device (Precision System Science, Chiba, Japan) and MagDEA DNA200 (Precision System Science). The purified DNA was adjusted to a concentration of 10 ng/μL using NanoDrop spectrophotometry for DNA amplification. The V3–V4 region of the 16S rDNA of gut microbiota prokaryotes was amplified using the universal primer set. The nucleotide sequencing of amplified DNA was then detected using the Illumina MiSeq sequencing system and MiSeq Reagent Kit v3 (Illumina, San Diego, CA, USA).

The partial sequence (approximately 380–430 bp) of the 16S rDNA was clustered using VSEARCH (version 2.4.3), setting the similarity threshold at 97%. Clusters that were identified with a confidence below 0.8 were predicted and grouped as unclassified. The classification was conducted using the Ribosomal Database Project (RDP) classifier. The number of each taxonomic group was calculated as the lead counts of the partial sequence of 16S rDNA. The relative abundance of each genus in the gut microbiota was calculated by dividing the read count of each genus by the total count. We used the relative abundance of each genus as the independent variable in regression analysis. IHPP measured a total of 413 gut microbiota at the genus level, and 232 genera were consistently testified from 2017 to 2019. Among them, we targeted the genera that have been reported to be associated with depression in previous research. Since the gut microbiota data at the species level was not measured in the IHPP, we can only go this far in our analysis.

### 2.3. The Center for Epidemiologic Studies Depression Scale

The Center for Epidemiologic Studies Depression Scale (CES-D), a renowned instrument for depression screening, was administered to participants of the IHPP. The CES-D comprises 20 items, each scored from 0 to 3, yielding a total score range of 0 to 60 [23]. Observations that yielded identical responses to all questions, such as assigning an “A” to every item, were excluded due to the presence of reverse-scored items in the CES-D. We dichotomized the CES-D scores, using a cutoff value of 16 points to signify the presence of depression. This dichotomized CES-D outcome was subsequently utilized as the dependent variable in our regression analysis.

### 2.4. Blood IL-6

The IHPP routinely collected whole blood samples from participants on an almost yearly basis. IL-6 levels were determined using a chemiluminescent enzyme immunoassay conducted by LSI Medience Corp (Tokyo, Japan) [24]. However, we did not have access to the blood IL-6 levels measured in 2019. Therefore, our regression analysis, which set IL-6 (pg/mL) as the dependent variable, was conducted exclusively using data from 2017 and 2018. We targeted IL-6 exclusively because other blood inflammatory markers like interleukin-1 were currently unavailable.

### 2.5. Soluble Fiber Intake

Our study aimed to illuminate the association between SFCAs, gut microbiota, and depression. However, blood/serum SFCA levels were unavailable in the IHPP. As a surrogate for SFCAs, we evaluated the monthly dietary intake of IHPP participants using the Brief-type self-administered Diet History Questionnaire (BDHQ), a tool specifically designed for Japanese adults [25]. From this, we calculated the daily intake of soluble fiber (g/day) for each participant. Given that soluble fiber serves as a substrate for butyrate production, we used daily soluble fiber intake as a proxy for SFCAs. This measure was then set as the dependent variable in our regression models.

### 2.6. Statistical Analysis

#### 2.6.1. Data Handling

The relative abundance of genera necessitated rescaling due to its original range from 0 to 1. To convert this into a continuous value that extends from negative infinity to positive infinity, we conducted a logit transformation on the relative abundance data. In order to prevent the generation of negative infinity following the logit transformation, we also added half of the minimum observed relative abundance value (excluding 0) to all observations.

Potential confounders, including grip strength (kg), gender, height (cm), weight (kg), smoking, and drinking habits, were incorporated into the models as covariates. For smoking and drinking statuses, participants were asked to select from one of three conditions: 1. Never had a smoking/drinking habit; 2. Currently smoking/drinking more than once per week; 3. Had a smoking/drinking habit previously. We subsequently recategorized these smoking and drinking statuses into two broader groups, namely, those who currently smoke/drink and all others.

#### 2.6.2. Regression Analysis

We employed the dichotomized CES-D (whether equal to or greater than 16), blood IL-6 concentration (pg/mL), and daily soluble fiber intake as dependent variables in respective regression analyses to investigate their associations with gut microbiota. For the participants who attended the IHPP health check-ups multiple times, data were aggregated using participant numbers, resulting in a dataset nested at the participant level. Therefore, we conducted regression analysis using generalized linear mixed models (GLMMs) with the participant ID set as a random effect [26]. When the dichotomized CES-D was set as the dependent variable, a logit-link function was utilized. In the case of continuous variables, such as IL-6 and daily soluble fiber intake, identical link functions were applied. Each gut microbiota genus’s relative abundance value was designated as the independent variable, resulting in a total of 37 regression models. Potential confounders, including grip strength (kg), gender, height (cm), weight (kg), smoking, and drinking habits, were incorporated as covariates in the GLMMs.

We also carried out regression analyses using GLMMs to estimate the associations of dichotomized CES-D with blood inflammatory marker IL-6 and daily soluble fiber intake, respectively. The previously introduced potential confounders were also included in these models.

#### 2.6.3. Multiple Testing Problem

Given that multiple statistical tests were conducted when estimating the regression coefficients, we performed corrections for multiple testing. We set the false discovery rate (FDR) at 0.05 to control the family-wise error rate and computed the adjusted *p*-value (q value). However, it is important to note that this study follows a purely observational design and does not directly impact medical decision-making. Therefore, the q values were provided for reference only. 

#### 2.6.4. Statistical Software

The open-source statistical software R version 4.2.2 and the lme4 packages developed for regression analysis using GLMM were utilized for data cleaning and analysis [27,28].

## 3. Results

### 3.1. Demographic Characteristics

Table 1 presents the demographic characteristics of the participants in our study. The results derived from 2192 observations are as follows: The mean age was 52.4 years, with a standard deviation of 15.2. The ratio of male to female was 956:1236, indicating a higher number of female participants in our analysis. The average height and weight were 162.1 cm and 60.9 kg, respectively. The mean grip strength was 31.1 kg. The average daily soluble fiber intake was noted to be 2.7 g. The proportion of current or former smokers was 15.3%. Additionally, 1070 participants reported either currently having a drinking habit or previously drinking more than once per week on a regular basis. Regarding depression prevalence, 778 participants reported CES-D scores equal to or greater than 16.

### 3.2. The Regression Analysis Outcomes

The outcomes of the regression analysis, which used GLMM to set the relative abundance value of each gut microbiota genus as the independent variable and the dichotomized CES-D as the dependent variable, are presented in Table 2. The regression coefficients of 11 gut microbiota genera, namely *Alistipes*, *Blautia*, *Coprococcus*, *Dorea*, *Faecalibacterium*, *Holdemania*, *Lactobacillus*, *Mitsuokella*, *Oscillibacter*, *Prevotella*, and *Roseburia*, demonstrated statistical significance. This finding suggests an association between the relative abundances of these gut microbiota and the presence of depression. The statistical significance of *Alistipes*, *Blautia*, *Coprococcus*, *Dorea*, *Faecalibacterium*, *Holdemania*, *Lactobacillus*, *Mitsuokella*, and *Oscillibacter* persisted even after correction for multiple testing, indicating that these associations are unlikely to be derived from Type I error. Among the gut microbiota genera with statistical significance, *Alistipes*, *Blautia*, *Coprococcus*, *Dorea*, *Faecalibacterium*, *Roseburia*, and *Oscillibacter* were reported to be associated with the butyrate-producing process [29,30,31,32,33].

We calculated the odds ratio (OR) for each gut microbiota genus and denoted the association as ↑ (positive, OR > 1) or ↓ (negative, OR < 1). Outcomes from previous studies involving either patients diagnosed with MDD or undiagnosed depression populations were also gathered and listed in Table 2. Inconsistencies in the associations of gut microbiota with depression within various studies may be attributable to differences in study location. For instance, the estimated OR of *Blautia* is 0.476, implying a negative association between the relative abundance of *Blautia* and the presence of depression. This outcome aligns with the results from two previous studies targeting the population living in Beijing, China [17,35]. Conversely, four prior studies involving participants from Chongqing, Hangzhou, and Taipei reported a positive correlation between the abundance of *Blautia* and depression [18,19,20,34]. Although these studies targeted residents in East Asia, from Beijing and Aomori prefecture, which share the same latitude, different latitudes of other areas may lead to variations in dietary habits, potentially resulting in discrepancies across these studies.

The outcomes of the regression analyses, utilizing GLMMs with IL-6 or daily soluble fiber intake as the dependent variables, are summarized in Table 3. The gut microbiota genera that demonstrated significant regression coefficients were *Erysipelotrichaceae incertae sedis* for IL-6, and *Anaerostipes*, *Collinsella*, *Faecalibacterium*, *Olsenella*, *Oscillibacter*, and *Parabacteroides* for daily soluble fiber intake. *Faecalibacterium*, known as a butyrate producer, exhibited a positive association (the regression coefficient equals 0.032) with soluble fiber intake, as expected. However, none of the coefficients maintained their significance after adjustment for multiple testing.

We also conducted a regression analysis using GLMM to estimate the association of the dichotomized CES-D with IL-6 and daily soluble fiber intake. Potential confounders, such as grip strength (kg), gender, height (cm), weight (kg), smoking, and drinking habits, were incorporated into the GLMM. The exponential regression coefficient, or in other words, the OR, for IL-6 and daily soluble fiber intake was 0.966 (*p* = 0.501) and 1.001 (*p* = 0.994), respectively. Given the absence of statistical significance for both, we did not proceed with the planned mediator analysis to determine how inflammatory markers and SCFAs might modify the association between gut microbiota and depression. 

## 4. Discussion

In our study targeting the Japanese population, we found that 11 gut microbiota genera, namely *Alistipes* (OR 0.910↓), *Blautia* (OR 0.476↓), *Coprococcus* (OR 0.796↓), *Dorea* (OR 0.764↓), *Faecalibacterium* (OR 0.870↓), *Holdemania* (OR 0.922↓), *Lactobacillus* (OR 1.112↑), *Mitsuokella* (OR 0.925↓), *Oscillibacter* (OR 0.830↓), *Prevotella* (OR 0.942↓), and *Roseburia* (OR 0.909↓) showed associations with the presence of depression. Even after multiple testing corrections, the statistical significance of *Alistipes*, *Blautia*, *Coprococcus*, *Dorea*, *Faecalibacterium*, *Holdemania*, *Lactobacillus*, *Mitsuokella*, and *Oscillibacter* remained unchanged. Prior research utilizing probiotic interventions has indicated that changes in the abundance of *Dorea*, *Lactobacillus*, and *Oscillibacter* are associated with a reduction in depression symptoms [45,46,47]. While more evidence from clinical trials and further discussions on causality are paramount, our findings align with these intervention studies and underscore the potential health benefits of microbial-targeted therapies for depression.

However, when IL-6 or daily soluble fiber intake was set as the dependent variable, the regression coefficient for each gut microbiota genus showed no statistical significance after adjustment for multiple comparisons. Additionally, no statistically significant coefficient was observed regarding the association of depression with IL-6 and daily soluble fiber intake.

As previously mentioned, gut microbiota composition is influenced by dietary habits. Among the bacterial genera that we found to be associated with depression in our study, *Coprococcus* has been reported to positively influence blood glucose fluctuations following high protein diets [48]. Consumption of processed meats was positively related to the abundance of *Holdemania* [49]. *Faecalibacterium* has also been reported to be associated with dietary habits [50]. Besides dietary habits, physical exercises may also influence gut microbiota composition. A previous clinical trial focusing on young adolescents reported an increase in the populations of *Blautia* and *Dorea* after a 3-month exercise intervention [51]. A majority of the bacterial genera significantly associated with depression in our study are involved in butyrate production. *Faecalibacterium prausnitzii*, the only known species of *Faecalibacterium*, has been reported as a main butyrate producer in the intestine [29]. Previous research has reported lower levels of Faecalibacterium in individuals with MDD [35,43], which aligns with our study result that the relative abundance of *Faecalibacterium* is negatively associated with the presence of depression. *Alistipes*, *Blautia*, *Coprococcus*, *Dorea*, *Roseburia* (although statistical significance was not observed after multiple testing corrections), and *Oscillibacter*, also known as butyrate-producing or inflammation-associated gut microbiota genera, were found to be negatively associated with the presence of depression [30,31,32,33]. Our results suggest that gut microbiota may influence depressive status via butyrate production.

It is unclear whether the association of gut microbiota with depression is influenced by inflammation and dietary habits. We had planned to perform a mediator analysis to clarify the roles of inflammation and dietary habits. However, our analysis using the IHPP dataset was unable to confirm the association of depression with IL-6 and daily soluble fiber intake, making the mediator analysis unfeasible. Moreover, even if the association had been confirmed, the IHPP data alone could not provide sufficient evidence to determine the causality between gut microbiota and depression due to the observational design of the study. Further evidence from intervention studies, including animal experiments and clinical trials, is needed to address this question. It is important to emphasize that the relationship between dietary habits and depressive status is likely intricate, involving numerous factors. Any assertions about causality between the two should be approached with caution to avoid overstating the influence of diet.

Our study has several limitations. First, given the observational nature of our design, we are unable to draw causal conclusions. However, future acquisition of more data from the IHPP may enable us to explore the temporal sequence of changes in gut microbiota composition and the onset of depression using a retrospective longitudinal design. Instead of a purely observational design, we are contemplating an intervention study that applies therapies targeting the microbiota for depression as a part of our future work. Second, extensive data on inflammatory markers and SCFAs is currently unavailable. Consequently, we could not perform regression analyses for inflammatory markers other than IL-6, and we had to use daily soluble fiber intake as a surrogate for intestinal SCFA concentrations. To clarify the influence of inflammatory markers and SCFAs on the relationship between gut microbiota and depression, we are considering incorporating additional blood tests for other inflammatory markers, such as C-reactive protein, and tests for intestinal SCFAs into samples from the IHPP. Third, the generalizability of our results may be limited because our study only focused on the Japanese population. Our goal was to provide evidence from an East Asian perspective. We believe that our findings, when combined with results from prior studies targeting other ethnic groups, contribute to a more comprehensive understanding of the relationship between gut microbiota and depression. Furthermore, the IHPP primarily recruited participants who were relatively healthy and could independently attend the health-check venue. This might introduce bias at the sampling stage, potentially affecting the generalizability of our results.

The present study uncovered associations between depression and several gut microbiotas in the Japanese population using the IHPP dataset, which has a relatively large sample size. The majority of these gut microbiotas contribute to the butyrate-producing process, underscoring the importance of butyrate. Further evidence, especially from intervention studies, is needed to unravel the mechanism by which these gut microbiota genera influence the depressive condition of the host.

## Figures and Tables

**Table 1:** The demographic characteristics of study participants.

Variables	Mean ± SD or Number (Proportion)
Age (year)	52.4 ± 15.2
Gender	Male 956 (43.6%); Female 1236 (56.4%)
Height (cm)	162.1 ± 8.9
Weight (kg)	60.9 ± 12.2
Grip strength (kg)	31.1 ± 9.3
Soluble Fiber Intake (g/daily)	2.7 ± 1.2
Current or ex-smoker (n)	336 (15.3%)
Current or ex-drinker (n)	1070 (48.8%)
Depression (CES-D score ≧ 16)	778 (35.5%)

For continuous variables, i.e., age, height, weight, grip strength, and soluble fiber intake, the mean and standard deviation are provided. Meanwhile, for categorical variables, including gender, presence of depression, and smoking and drinking habits, the count number and percentage are displayed.

**Table 2:** Association between gut microbiota and depression.

Gut Microbiota	Odds Ratio	*p*-Value	q-Value	Results from Previous Studies	
				Targeting MDD	Targeting Depression
*Eubacterium*	1.007↑	0.915	0.915	↑[34], ↑[25]	
*Paraprevotella*	0.993↓	0.851	0.875	↑[21]	↑[35]
*Escherichia.Shigella*	0.991↓	0.812	0.858	↓[18]	↓[35]
*Bacteroides*	0.971↓	0.757	0.823	↑[34], ↑[36], ↓[18], ↓[37], ↓[38], ↓[20]	↑[35]
*Veillonella*	0.978↓	0.721	0.808	↑[34]	↑[35]
*Desulfovibrio*	0.970↓	0.694	0.803	↑[34], ↑[36], ↑[38]	
*Enterococcus*	0.983↓	0.645	0.770		↑[39]
*Bifidobacterium*	0.978↓	0.636	0.770	↑[34], ↑[19], ↑[37], ↑[38], ↓[40]	↓[35]
*Streptococcus*	1.035↑	0.555	0.708	↑[19], ↑[41], ↑[38], ↑[20]	
*Erysipelotrichaceae* *incertae sedis*	1.058↑	0.453	0.598	↑[34], ↑[19], ↑[18], ↑[20]	
*Clostridium.XlVa*	0.932↓	0.436	0.597	↓[20]	
*Sutterella*	0.978↓	0.428	0.597	↓[34], ↓[19], ↓[20]	↓[35]
*Clostridium.XI*	0.926↓	0.405	0.597	↑[19], ↑[41]	↓[35]
*Parabacteroides*	0.935↓	0.400	0.597	↑[19], ↑[18]	↑[42], ↓[35]
*Dialister*	0.971↓	0.396	0.597	↓[18], ↓[21]	↑[35], ↓[43]
*Anaerostipes*	0.949↓	0.392	0.597	↑[34], ↑[20]	
*Butyricimonas*	0.960↓	0.347	0.597	↑[18]	↓[35]
*Eggerthella*	0.949↓	0.304	0.563	↑[34], ↑[19], ↑[21], ↑[37], ↑[38], ↑[20]	
*Haemophilus*	0.959↓	0.291	0.563	↓[18], ↓[38]	↑[35]
*Collinsella*	0.948↓	0.289	0.563		↑[20]
*Flavonifractor*	0.904↓	0.207	0.449	↑[34], ↓[17], ↓[18], ↓[20]	↓[35], ↓[43]
*Phascolarctobacterium*	0.950↓	0.136	0.315	↑[18], ↓[20]	↑[43]
*Olsenella*	1.167↑	0.122	0.300	↑[34], ↑[37], ↑[20]	
*Subdoligranulum*	0.831↓	0.104	0.275	↓[44]	
*Ruminococcus*	0.952↓	0.053	0.151	↑[19], ↓[18]	↓[35]
*Megamonas*	0.897↓	0.051	0.151	↑[18], ↓[19], ↓[20]	↓[35]
*Prevotella*	0.942↓	0.025 *	0.084	↑[41], ↓[19], ↓[18], ↓[21]	↑[35]
*Roseburia*	0.909↓	0.015 *	0.054	↑[34], ↑[19]	↓[35]
*Faecalibacterium*	0.870↓	0.006 *	0.026 *	↑[34], ↓[17], ↓[18], ↓[20]	↓[35], ↓[43]
*Lactobacillus*	1.112↑	0.005 *	0.022 *	↑[37], ↑[38], ↑[20]	↑[43]
*Blautia*	0.476↓	0.002 *	0.012 *	↑[34], ↑[19], ↑[18], ↑[20], ↓[17]	↓[35]
*Dorea*	0.764↓	0.000 *	0.000 *	↑[20], ↓[17]	
*Coprococcus*	0.796↓	0.000 *	0.000 *	↓[17], ↓[20]	↓[35], ↓[43]
*Holdemania*	0.922↓	0.000 *	0.000 *	↑[19], ↑[21]	↑[43]
*Oscillibacter*	0.830↓	0.000 *	0.000 *	↑[18], ↑[37], ↑[38]	↓[35]
*Alistipes*	0.910↓	0.000 *	0.000 *	↑[18], ↓[20]	↑[35]
*Mitsuokella*	0.925↓	0.000 *	0.000 *		↓[35]

MDD stands for Major Depressive Disorder. The * mark indicates statistical significance. According to the current data from IHPP, 11 gut microbiota genera are associated with depression, and among them, the association with nine genera remains unchanged even after multiple testing corrections. Associations are denoted by the ↑ mark for a positive relationship and the ↓ mark for a negative relationship.

**Table 3:** Association between gut bacteria, IL-6, and soluble fiber intake.

Gut Microbiota	IL-6			Soluble Fiber Intake		
	Beta	*p*	q	Beta	*p*	q
*Alistipes*	−0.041	0.298	0.798	−0.015	0.124	0.417
*Anaerostipes*	−0.008	0.907	0.948	0.041	0.008 *	0.138
*Bacteroides*	−0.093	0.383	0.798	−0.020	0.425	0.585
*Bifidobacterium*	0.087	0.084	0.798	0.010	0.399	0.585
*Blautia*	−0.128	0.310	0.798	0.042	0.178	0.483
*Butyricimonas*	−0.015	0.744	0.888	−0.015	0.209	0.483
*Clostridium.XI*	−0.018	0.829	0.930	0.021	0.277	0.506
*Clostridium.XlVa*	0.038	0.703	0.888	−0.037	0.112	0.413
*Collinsella*	0.058	0.080	0.798	−0.021	0.010 *	0.138
*Coprococcus*	0.016	0.625	0.826	0.007	0.396	0.585
*Desulfovibrio*	−0.048	0.537	0.798	−0.005	0.791	0.887
*Dialister*	−0.016	0.612	0.826	−0.007	0.371	0.585
*Dorea*	−0.004	0.923	0.948	0.001	0.894	0.918
*Eggerthella*	0.033	0.530	0.798	−0.022	0.084	0.390
*Enterococcus*	−0.024	0.553	0.798	0.009	0.301	0.506
*Erysipelotrichaceae incertae sedis*	−0.098	0.045 *	0.798	−0.015	0.203	0.483
*Escherichia.Shigella*	0.002	0.948	0.948	−0.010	0.241	0.499
*Eubacterium*	−0.053	0.418	0.798	−0.027	0.061	0.325
*Faecalibacterium*	−0.051	0.331	0.798	0.032	0.012 *	0.138
*Flavonifractor*	−0.051	0.486	0.798	−0.020	0.243	0.499
*Haemophilus*	−0.055	0.197	0.798	0.008	0.427	0.585
*Holdemania*	−0.038	0.516	0.798	−0.003	0.829	0.890
*Lactobacillus*	−0.026	0.498	0.798	−0.004	0.629	0.776
*Megamonas*	−0.004	0.893	0.948	−0.002	0.842	0.890
*Mitsuokella*	0.058	0.272	0.798	−0.001	0.918	0.918
*Olsenella*	−0.043	0.509	0.798	−0.031	0.046 *	0.286
*Oscillibacter*	0.040	0.455	0.798	−0.032	0.015 *	0.138
*Parabacteroides*	0.030	0.549	0.798	−0.028	0.023 *	0.170
*Paraprevotella*	−0.020	0.561	0.798	−0.012	0.197	0.483
*Phascolarctobacterium*	0.009	0.782	0.904	−0.009	0.298	0.506
*Prevotella*	−0.010	0.721	0.888	−0.011	0.110	0.413
*Roseburia*	−0.034	0.468	0.798	0.014	0.201	0.483
*Ruminococcus*	−0.032	0.237	0.798	−0.002	0.741	0.874
*Streptococcus*	−0.051	0.435	0.798	0.008	0.584	0.745
*Subdoligranulum*	0.130	0.279	0.798	−0.015	0.562	0.743
*Sutterella*	−0.020	0.525	0.798	0.009	0.267	0.506
*Veillonella*	−0.058	0.136	0.798	0.003	0.756	0.874

The * symbol denotes the statistical significance. Setting blood IL-6 as the dependence variable in the GLMM, only the regression coefficient of *Erysipelotrichaceae incertae sedis* showed statistical significance. The relative abundance of *Anaerostipes*, *Collinsella*, *Faecalibacterium*, *Olsenella*, *Oscillibacter*, and *Parabacteroides* were found be associated with daily soluble fiber intake. Though the statistical significance is absent after multiple testing corrections.

## Data Availability

Restrictions apply to the availability of these data. Data were obtained from the Hirosaki Center of innovation and are available at https://coi.hirosaki-u.ac.jp/en/ (accessed on 3 July 2023) with the permission of the Hirosaki Center of innovation.
